# Sialidosis: A Review of Morphology and Molecular Biology of a Rare Pediatric Disorder

**DOI:** 10.3390/diagnostics8020029

**Published:** 2018-04-25

**Authors:** Aiza Khan, Consolato Sergi

**Affiliations:** 1 Department of Laboratory Medicine and Pathology (5B4.09), University of Alberta, 8440 112 St NW, Edmonton, AB T6G 2B7, Canada; draizakhan@gmail.com; 2 Department of Pediatrics, Stollery Children’s Hospital, University of Alberta Hospital, Edmonton, AB T6G 2B7, Canada

**Keywords:** sialidosis, neuraminidase, sialidosis I, sialidosis II, lysosomal storage disease, lysosomal exocytosis

## Abstract

Sialidosis (MIM 256550) is a rare, autosomal recessive inherited disorder, caused by α-*N*-acetyl neuraminidase deficiency resulting from a mutation in the neuraminidase gene (*NEU1*), located on 6p21.33. This genetic alteration leads to abnormal intracellular accumulation as well as urinary excretion of sialyloligosaccharides. A definitive diagnosis is made after the identification of a mutation in the *NEU1* gene. So far, 40 mutations of *NEU1* have been reported. An association exists between the impact of the individual mutations and the severity of clinical presentation of sialidosis. According to the clinical symptoms, sialidosis has been divided into two subtypes with different ages of onset and severity, including sialidosis type I (normomorphic or mild form) and sialidosis type II (dysmorphic or severe form). Sialidosis II is further subdivided into (i) congenital; (ii) infantile; and (iii) juvenile. Despite being uncommon, sialidosis has enormous clinical relevance due to its debilitating character. A complete understanding of the underlying pathology remains a challenge, which in turn limits the development of effective therapeutic strategies. Furthermore, in the last few years, some atypical cases of sialidosis have been reported as well. We herein attempt to combine and discuss the underlying molecular biology, the clinical features, and the morphological patterns of sialidosis type I and II.

## 1. Introduction

Sialidosis, an autosomal recessive disorder, occurs due to a structural defect in the neuraminidase gene and is characterized by abnormal tissue accumulation as well as urinary excretion of sialylated oligosaccharides and glycolipids [1].

The human neuraminidase gene is located at chromosome band 6p21.3, where the *HLA* locus is also reported to be located [2]. Until 1977, deficiency of Neuraminidase 1 (NEU1) was thought to be associated with classical mucolipidosis I, a severe and rapidly progressive lysosomal storage disease with onset at birth or shortly after birth [3,4]. In 1977, the term sialidosis was first used to describe the syndrome of two siblings having a visual impairment and mild neurological manifestations that slowly developed in their adolescence. Enzymatic assays in cultured fibroblasts and leukocytes from these siblings exhibited an isolated deficiency of NEU1 [3,4]. Later, Sialidosis was classified into two types: Sialidosis Type I (normomorphic) and Sialidosis Type II (dysmorphic) [5]. In this study, we review the underlying genetics and molecular mechanisms of sialidosis and their correlation with clinical and morphological findings.

## 2. Background

Sialidase (neuraminidase, EC 3.2.1.18) catalyzes the hydrolysis of terminal sialic acid residues of glycoconjugates. Sialidase has been extensively studied in viruses and bacteria. In these microorganisms, its function is to destroy the sialic acid-containing receptors at the surface of host cells to mobilize bacterial nutrients. In mammals, three types of sialidases have been reported, including the lysosomal, plasma membrane, and cytosolic localized enzymes [1].

In human lysosomes, the degradation of complex macromolecular substrates requires the synergistic action of multiple hydrolases that act synergistically to carry out the degradation process of complex macromolecular substrates efficiently. One such efficient catalytic team is formed by three hydrolases which are ubiquitous, but differentially expressed: the serine carboxypeptidase, protective protein/cathepsin A (PPCA), the sialidase, Neuraminidase-1 (NEU1), and the glycosidase β-Galactosidase (β-GAL) [6]. The different expression of three enzymes can be explained by the fact that deficiency of each leads to three distinct lysosomal storage disorders (LSDs): galactosialidosis (GS) or PPCA deficiency with a secondary combined deficiency of NEU1 and β-GAL, sialidosis or NEU1 deficiency, and GM1-gangliosidosis (GM1) or β-GAL deficiency. Each disease is inherited as an autosomal recessive trait and is distinguished by variable clinical phenotypes, ranging from congenital forms to infantile/juvenile forms. All three present as a systemic disease, involving visceral organs, bone, cartilage, muscle and the nervous system [6].

Catalytic activation of NEU1 is PPCA dependent: The function of NEU1 is to initiate the catabolism glycoproteins and glycolipids, by cleaving their terminal sialic acids. For this, NEU1 depends on its interaction with the auxiliary protein protective protein/cathepsin A (PPCA). PPCA is essential for the stability of NEU1 and acts as a molecular chaperone for the subcellular localization and compartmentalization [7]. It can be assumed that NEU1 mutations that affect its interaction with PPCA may also lead to disease, even if the residues forming the active site of the enzyme remain intact [8]. In fact, researchers using the crystal structures of bacterial sialidases as templates have investigated numerous NEU1 amino acid substitutions related to different clinical phenotypes. Most of those substitutions appear to be located at the core surface of the molecule, demonstrating that they may affect the interaction of NEU1 with its chaperone PPCA. Three pathogenic mutations, F260Y, L270F, and A298V, gathered at the surface of the bacterial sialidases. These enzymes were correctly synthesized yet degraded instantly since the resulting proteins failed to associate with PPCA [9]. A putative region of interaction between NEU1 and PPCA was noticed after a careful study of the hydrodynamic properties of these two proteins. This region appeared to be crucial for NEU1 binding to the precursor form of PPCA. Therefore, *NEU1* mutations affecting amino acids within this domain may affect the stability of the enzyme and subsequent PPCA-mediated transportation to lysosomes [7,8,9]. It is crucial to remember that a primary defect of PPCA results in the lysosomal disorder galactosialidosis [2]. The similarity in the clinical symptoms of these two disorders can be attributed to the fact that the absence of functional PPCA results in a near-complete secondary deficiency of NEU1 [10]. 

**Figure 1:**
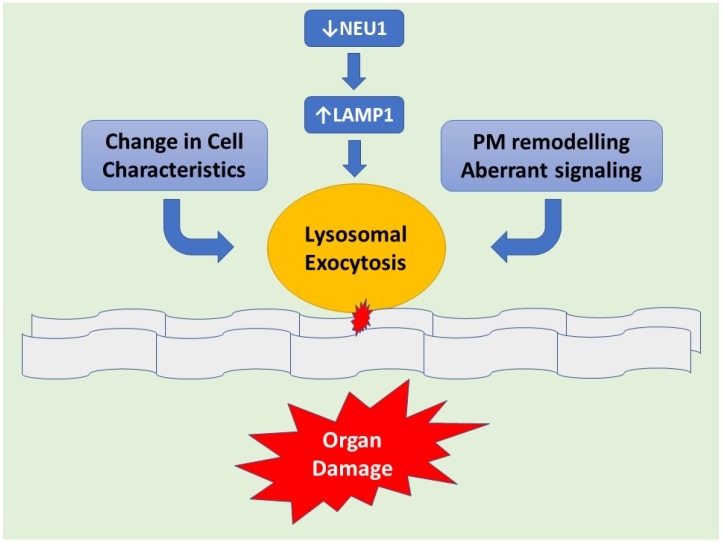
Schematic representation demonstrating downstream of NEU1 deficiency leads to LAMP1 accumulation causing an increased number of lysosomes at the plasmatic membrane (PM) resulting in exacerbated lysosomal exocytosis. Lysosomal-associated membrane protein 1 (LAMP1), aka lysosome-associated membrane glycoprotein 1 or CD107a, is a protein that in humans is determined by the *LAMP1* gene. This abnormal release of lysosomal content causes extracellular PM remodeling. Hence changes in cell characteristics take place with subsequent organ pathogenesis.

Mechanisms of Pathogenesis in Sialidosis explained with the help of the Mouse Model: The *Neu1*−/− knockout (KO) mouse model is helpful in understanding the underlying molecular mechanism(s) of sialidosis. *Neu1*−/− mice demonstrated NEU1 as a negative regulator of lysosomal exocytosis. It was observed that in hematopoietic cells, NEU1 negatively regulates lysosomal exocytosis by processing the sialic acids on the lysosomal membrane protein 1 (LAMP1). LAMP1 is an integral membrane protein and plays a useful role in the docking of lysosomes at the plasma membrane (PM). Deficiency or impaired NEU1 activity causes defective processing of the sialic acids on LAMP1, causing accumulation of LAMP1 in an over-sialylated state with a prolonged half-life. This accumulation of over-sialylated LAMP1 increases the number of LAMP1-marked lysosomes that dock at the PM, poised to engage in lysosomal exocytosis upon an influx of calcium. LAMP1’s essential function in the docking of lysosomes to the PM has been further supported by the fact that silencing *LAMP1* in NEU1-deficient cells normalize the number of lysosomes docked at the PM and thus decreased the extent of lysosomal exocytosis. Hence, NEU1 loss of function ultimately results in the excessive extracellular release of lysosomal luminal contents from deficient cells of several tissues and organs (Figure 1). Excessive lysosomal exocytosis has now been associated with various pathological manifestations which are characteristic of sialidosis, including its role in neurodegeneration and links with Alzheimer’s disease, hearing loss, muscle atrophy and splenomegaly [11]. Moreover, phagocytosis in macrophages, which is regulated by NEU1, as well as NEU1-dependent regulation of insulin signaling are the other pathways that have been considered as additional pathological mechanisms involved in the NEU1 loss of function of the organs [12].

## 3. Morphological and Clinical Aspects of Sialidosis and *NEU1* Mutation(s)

### 3.1. Method of Study Selection, Criteria, and Data Extraction

Initially, an electronic search was done on Google Scholar, Scopus, and PUBMED. The search was conducted between the time of January 1980 to January 2018. The words used were sialidosis I, sialidosis II, congenital sialidosis, infantile sialidosis, juvenile sialidosis and combinations of these words. In the next step, reference lists of the publications were checked to identify any additional studies. The search was limited to studies published in English in the open literature in peer-reviewed journals. 

Cases of sialidosis I typically present later in life (second to third decade); therefore, sialidosis I was discussed briefly in this study. However, for sialidosis II, all cases found were included in the paper since this condition is present in the pediatric group of patients.

Sialidosis (MIM #256550) is known as an autosomal recessive inherited disease. However numerous times, genetic alterations have been found in *NEU1* of unrelated sialidosis patients. So far, more than 40 mutations within the *NEU1* gene have been identified in patients with sialidosis types I and II. Age of onset and severity of the clinical manifestations are paralleled with *NEU1* mutations and the level of residual neuraminidase activity, demonstrating the existence of significant genotype-phenotype correlation in sialidosis.

NEU1 protein variants have been categorized into three groups based on biochemical properties. In the first group, the mutant enzyme stays catalytically inactive and does not localize to the lysosomes; whereas in the second group, the mutant protein localizes to the lysosomes yet is enzymatically inactive. Finally, in the third group, the mutant protein has residual activity and localizes to the lysosomes. An association appears to exist between individual mutations and the clinical severity of sialidosis [7]. In the least severe form of sialidosis, the modification is thought to cause a decrease in sialidase activity. However, mutant sialidase has residual activity as well as localizing to the lysosomes. Hence, leading to sialidosis I.

### 3.2. Sialidosis I

Also known as cherry-red spot myoclonus syndrome, type I sialidosis is the less severe, non-neuropathic subtype of this disease. Patients typically exhibit symptoms in the second or third decade of life. Symptoms may include gait abnormalities, decreased visual acuity, or both. Patients usually have no physical defects. Their intelligence level may range from normal to slightly impaired [13]. Myoclonus is an essential feature of sialidosis I, which over the course of the disease tends to get disabling. Precipitating factors may include light touch, sound stimuli, voluntary movements, passive joint movements, voluntary movements, and dysarthria. Action myoclonus, intentional tremors, cerebellar ataxia, and hyperreflexia are the other commonly found symptoms [14]. Muscle strength may remain normal. However, hypotonia can be seen. In some cases, the patients may become wheelchair-bound as the disease progresses [7]. Laboratory tests for sialidosis include a thin-layer chromatography test that is a useful screening test to find an abnormal urinary oligosaccharide pattern. Peripheral blood smear or bone marrow smear may show the presence of storage granules in lymphocytes. Deficiency of the lysosomal sialidase activity (neuraminidase) can be demonstrated in cultured skin fibroblasts obtained from a skin biopsy and is an important diagnostic step. Importantly, in enzymatic studies, sialidosis is differentiated from galactosialidosis by analyzing the enzymatic activity of β-galactosidase which should be normal. The final diagnosis is made after whole genome sequencing. As mentioned earlier, symptoms and their extent of severity are closely associated with the type of *NEU1* mutations involved and subsequently the levels of residual enzyme activity [15]. 

Pathologically, cytoplasmic accumulation of sialyloligosaccharides has been observed in many neurons in the central nervous systems (CNS) of sialidosis patients. Moreover, neuroradiological imaging studies frequently reveal diffuse brain atrophy in the advanced stage of sialidosis type I, particularly of the cerebellar area [16]. However, initial neuroradiological investigations can be unremarkable in sialidosis 1. Studies also suggest that the major clinical effects seen are caused by changes at a level above the brainstem [17,18,19,20]. Further investigation in this area will help in understanding the underlying pathological mechanism which will consequently lead to the availability of better therapeutic approaches.

#### Atypical Cases of Sialidosis I

In the last decade, one crucial observation regarding sialidosis I is the presence of isolated instances of action myoclonus. Myoclonus, which is considered an essential feature of sialidosis I, has been seen in patients in the absence of other classic symptoms (macular cherry-red spot and sialyloligosacchariduria). After whole genome sequencing, mutations in *NEU1* were identified. This aspect elucidates the fact that mutations affecting NEU1 activity can exist in the absence of other clinical signs that are characteristic of sialidosis [21]. This aspect warrants further studies in this area to investigate if there are more cases of sialidosis I than initially predicted.

### 3.3. Sialidosis II

Based on the age at onset of the symptoms, type II sialidosis is further divided into three subtypes: (i) congenital or hydropic (in utero); (ii) infantile (0–12 months); and (iii) juvenile (2–20 years) [7,22].

**Figure 2:**
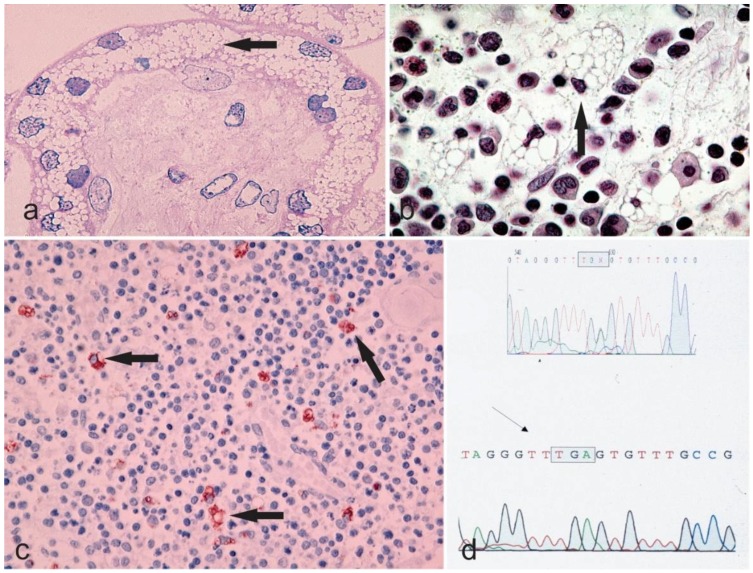
Microphotographs **a**–**c** showing the vacuolar degeneration of syncytium-trophoblast of a placenta (Hematoxylin & Eosin staining, 400×), bone marrow (Hematoxylin & Eosin staining, 630×, the arrow points to a cell with margination of the nucleus due to an engulfment of the cytoplasm with undigested material), and thymus (Anti-CD68 immunostaining, 200× with the arrows highlighting the macrophages) from a sialidosis pregnancy. The chromatogram in **d** shows the genetic alteration of the sialidosis gene.

The congenital or hydropic subtype: Profoundly severe mutational alterations may lead to a complete absence of lysosomal neuraminidase and are lethal during fetal development or at birth. The congenital type of sialidosis, which is the severe form of the disease, is thought to be the result of such mutations. It manifests itself prenatally and is characterized by ascites and hydrops fetalis, hepatomegaly and stillbirths or death at a very early age [7]. Table 1 summarizes the cases of congenital sialidosis reported from 1979 until now (cases presented before 1979 can be found in a previous review [5]). It can be observed that hydrops, ascites, and edema are the distinguishing features of the severe, congenital group of the disease, followed by coarse features, dysostosis multiplex, and hepatosplenomegaly. Renal involvement, cardiac anomalies, ophthalmic finding, myoclonus, inguinal hernia, telangiectasias, petechiae, bluish to purpuric macules and hydrocephalus are the clinical features that may infrequently manifest [9,22,23,24,25,26,27,28,29,30,31,32,33,34,35,36,37,38,39,40,41,42,43,44,45,46]. Studies reporting histopathological features of congenital sialidosis are limited. In one study, light microscopy of fetal tissues (after pregnancy was terminated at 20 weeks) exhibited vacuolation in the liver, bone marrow, kidney, and brain. In addition, an abnormal pattern of vacuolations was also present in the endocrine organs such as the thyroid gland, adrenal gland, hypophysis, testes as well as in the thymus. Moreover, vacuolation of the placenta demonstrated that in congenital sialidosis abnormal storage takes place during the early fetal period [36]. Figure 2 illustrates vacuolation of placenta, spleen, and thymus of a patient with congenital sialidosis.

Infantile/juvenile subtype: It has been suggested that *NEU1* mutations in which mutant enzymes localize to the lysosomes yet stay enzymatically inactive lead to the infantile/juvenile subtype of sialidosis II, which is characterized by the development of progressive mucopolysaccharidosis-like phenotype; Patients presenting with coarse facies, visceromegaly, dysostosis multiplex, vertebral deformities, mental retardation [7,47,48,49,50,51,52,53,54,55]. Table 2 summarizes the clinical features of the infantile/juvenile subtype. Skeletal abnormalities, mainly dysostosis multiplex appear to be a consistent feature of infantile/juvenile phenotypes, along with coarse facies, hepatosplenomegaly, and severe mental retardation. Ocular manifestations, including cherry red spots, cataracts, nystagmus, strabismus, and corneal clouding are common as well while hypotonia, renal involvement, and cardiac anomalies are relatively infrequent findings. It is noticeable that earlier onset of disease has a fulminant course. While in late-onset, the patient may survive for more extended periods (only four patients survived for more than two decades). Hearing loss and ataxia may present in the late onset of infantile/juvenile form and tend to worsen with time. One case of sialidosis II was reported in the last decade, in which the patient developed myoclonic seizures at the age of 17, followed by dysphagia and dysphonia. There was a marked delay in motor and cognitive functions since childhood which worsened over time. However, the patient was able to achieve an elementary school education. At the time of diagnosis, the patient was 30 years old and bedridden, with an advanced degree of mental deficiency. It is notable that the patient was able to achieve education demonstrates that her cognitive delay was not as severe in childhood but progressed with age [55]. This detail seems essential and may aid in future developing therapeutic interventions in this area. Also, it is noteworthy that myoclonus, which is considered a characteristic of sialidosis I, is found in both Congenital as well as Infantile/Juvenile subtypes. (Table 1 and Table 2).

NEU1 knockout model *Neu1*−/− mice developed a systemic and neurodegenerative condition that is comparable to the early onset of sialidosis II [10] thus helping in understanding the clinical manifestation of this disease. Some typical clinical features that have been studied and explained are as follows. Hepatosplenomegaly is a fairly standard feature in sialidosis II. Enlargement of the spleen observed in *Neu1*−/−mice is consistent with this finding. Histological studies of spleens in mutant mice showed a time-dependent expansion of total splenic cell counts along with an increased number of erythroid precursors and megakaryocytes. Along with these changes in the spleen, an increased number of hematopoietic progenitors in the peripheral blood and an overall lower number of these cells in the bone marrow (BM) was found. Thus, indicating extramedullary hematopoiesis (EMH) as the possible cause of splenic hypertrophy in *Neu1*−/−. Furthermore, immunohistochemical studies of mutant livers showed a high number of erythroblasts, suggesting extramedullary hematopoiesis in this organ as well. Pathological studies of livers revealed initial ballooning followed by progressive, age-dependent filling of sinusoidal cells and hepatocytes with vacuoles [10]. Skeletal abnormalities frequently occur in sialidosis II. According to researchers, a possible hypothesis explaining the underlying pathogenesis is the impaired function of osteoclasts resulting in inefficient bone remodeling and consequently leading to bone deformities [10]. Hearing loss is another feature of sialidosis II that appears late in the disease. Studies of the (KO)model showed both conductive and neurosensory defects contribute to hearing loss in *Neu1*−/− mice. It has been suggested that the absence of NEU1 and the potential exacerbation of lysosomal exocytosis in the inner ear are responsible for the occurrence of hearing the loss in *Neu1*−/− mice. Histopathological studies exhibited thickened, cerumen occlusion in the external auditory canal, while in the middle ear infiltration of connective tissue with signs of chronic inflammation was noticed. Also, in many cells of the extensive cochlea vacuolization was seen [56]. In the brain, *Neu1*-null mice show extensive vacuolization of the epithelial cells of the choroid plexus and the endothelial cells of the ependymal layer 31, 33. Microglia and perivascular macrophages were among the most affected cells. This feature was prominently noticeable in the dentate gyrus and the hippocampus, but these cells are also scattered throughout the cortex and in the cerebellum, causing a widespread microgliosis [10]. Additionally, in the hippocampal region of the *Neu1*-null mice, recent studies have suggested a link between NEU1 deficiency-exacerbated lysosomal exocytosis and the spontaneous occurrence of Alzheimer’s disease (AD)-like amyloidogenic process. Essentially accumulation in endo-lysosomes of an over sialylated amyloid precursor protein (APP), which is a newly identified substrate of NEU1, initiates the process. Next Endo-lysosomal APP is proteolytically cleaved to generate amyloid β-peptide isoforms (Aβ), which is ultimately released extracellularly by excessive lysosomal exocytosis. The finding that intracranial injection of NEU1 in the AD mouse model reduces the numbers of amyloid plaques and the levels of amyloid peptides is exceptionally important, as it demonstrates that NEU1 can be explored as a therapeutic approach for AD [57].

## 4. Therapeutic Interventions for Sialidosis

Due to the rarity of the disease, establishing optimum therapeutic measures remains a challenge although many promising methods are being proposed and being investigated. One attempt of enzyme replacement therapy (ERT) in *Neu1*−/− mice using a recombinant NEU1 enzyme purified from overexpressing insect cells was made, which helped significantly in increasing levels of the NEU1 protein, and this treatment achieved subsequent correction of the underlying pathology in most of the systemic organs. However, the recombinant protein turned out to be highly immunogenic in the mutant mice and thus causes a severe immune response. Consequently, the therapeutic use of ERT became restricted [58,59]. Another study investigated the efficacy of the immuno-suppressant (Celastrol) along with a proteasomal inhibitor (MG132) as a therapeutic option for sialidosis. Researchers found that MG132 enhances enzyme activity and its localization in cells expressing defective sialidase provided promising results [60]. Additionally, chaperone-mediated gene therapy using a new mouse model has been suggested. The new mouse model ubiquitously expresses a NEU1 variant, which has a V54M amino acid substitution found in an adult patient with type I sialidosis. Mutant mice exhibited signs of lysosomal disease after one year of age, with low residual NEU1 activity detected in most organs and cell types. Injection of aged mutant mice with AAV-PPCA caused improvement of symptoms in the disease phenotype, hence suggesting that some NEU1 mutations associated with type I sialidosis may respond to PPCA-chaperone-mediated gene therapy [61]. Researchers believe that the treatment may be useful for other NEU1 mutations, particularly those in patients with type I sialidosis [8].

## 5. Conclusions and Future Perspectives

Sialidosis is an autosomal recessive disease resulting due to a mutation in the neuraminidase (*NEU1*) gene. Missense mutations appear to be the most commonly occurring. Among these mutations, a significant molecular heterogeneity is present, with a mixed variety of clinical phenotypes presenting either as sialidosis I or sialidosis II with different levels of severity. The onset of disease, the severity of symptoms, and prognosis differ in sialidosis I and sialidosis II. Bonten et al. demonstrated that this association between the clinical phenotype and severity of the disease is the result of the residual activity of the mutant enzymes. Patients with type II disease have catalytic inactive neuraminidase, while patients with the mild type I disease have active catalytic neuraminidase. In the type I disease, there is an absence of any obvious physical defects, and life expectancy remains unaffected. However, progressive visual loss and myoclonus, which may tend to get worse with time, can be disabling. In type II, the congenital subtype takes a fulminant course with very low life expectancy. Ascites and edema are the prominent features of this subgroup of the disease. In the infantile/juvenile sub type, characteristic features include hepatosplenomegaly, dysostosis multiplex, coarse facies, cherry-red spot, myoclonus, and severe mental retardation. Some clinical manifestations such as ataxia and hearing loss may become progressively severe with age. In the last decade, atypical cases of sialidosis 1 have been reported, and this aspect warrants further studies in this area to investigate if there are more cases of sialidosis I than initially predicted. Furthermore, the possibility suggested by previous researchers that, in addition to neuraminidase mutations, environmental factors including diet, prophylactic therapies, or other genetic factors may have some effect on the penetrance and severity of the disease and/or phenotype of the disease seems more plausible [7]. Perhaps in the future, a more detailed prenatal history inquiring about the diet, medication, and different types of environmental exposure of parents may help the researchers understand this aspect of the disease. Therapeutic options for sialidosis remain limited. Researchers have emphasized exploring treatment options for sialidosis I [8]. In sialidosis I, symptoms are relatively mild and appear late. Although intelligence level remains normal, myoclonus can be debilitating. Therefore, effective therapy may improve quality of life. In sialidosis type II patients, an early onset, systemic involvement, and a fulminant course make it more challenging to provide treatment. Perhaps it would be worthwhile to explore the suggestion of taking an in utero approach for therapy in the future. Alternatively, carrier detection in affected families, prenatal molecular diagnosis, and improved genetic counseling seem to be a suitable practical approaches. Finally, NEU1 appears to have a crucial link with the CNS. Studies provide substantial evidence to demonstrate the NEU1 enzyme has a correlation with Alzheimer’s disease. Further understanding of NEU1 and its link with the CNS may provide useful information regarding genetics, pathogenesis, and treatment of not only sialidosis, but also Alzheimer’s disease.

## Figures and Tables

**Table 1:** Clinical and Morphological findings of patients suffering from sialidosis II, congenital subtype form. (Cases from 1980 until present are included). M = male; F = female; n/a = not available; n/r = not reported; no = not detected.

References (Name of the First Author)	Gender	General Presentation	Nervous System	Ophthalmologic Findings	Skeleton	Respiratory Distress/Infections	Renal Involvement	Cardiac Involvement	Others	Course of Disease
Kelly, T.E. [23]	F	Ascites, edema, hepatosplenomegaly course features	n/r	n/r	Dysostosis multiplex	n/r	n/r	Cardiac anomalies	n/r	Exitus at 26 months
Riches, W.G. [24]	F	Hydrops, ascites, hepatosplenomegaly	n/r	n/r	n/r	n/r	n/r	n/r	n/r	Exitus at 4 months
Gillan, J.E. [25]	M	Hydrops, ascites, edema, hepatosplenomegaly course features	n/r	n/r	n/r	n/r	n/r	n/r	n/r	Exitus at 3 days
Beck, M. [26]	F	Hydrops, ascites, edema, hepato-splenomegaly, coarse features	n/r	n/r	n/r	present	present	n/r	n/r	Exitus at 6 months
Guibaud, P. [27]	F	Hydrops, ascites, edema, hepato-splenomegaly, coarse features	n/r	Corneal clouding	Dysostosis multiplex	n/r	n/r	n/r	n/r	n/r
Johnson, W.G. [28]	M M F F	Hydrops, Ascites, edema	Seizures	n/r	n/r	n/r	n/r	n/r	Telangiectasia	stillborn, 1 month, 3 months, alive 3 months
Yamano, T. [29]	M	Hydrops, ascites, edema, hepato-splenomegaly	n/a	no	no	n/a	n/a	n/a	n/a	Exitus at 56 days
Tabardel, Y. [30]	n/r	Hydrops, ascites, coarse features, hepatosplenomegaly	n/r	n/r	n/r	n/r	n/r	Cardiac anomalies	Petechiae	n/r
Ries, M. [31]	n/a	hydrops, ascites	n/r	Cherry red spots	n/r	n/r	n/r	n/r	n/r	Exitus at 28 days
Lukong, K.E. [9]	F	hydrops	n/r	n/r	n/r	n/r	n/r	n/r	n/r	Exitus at 82 days
Nakamura, Y. [32]	F	Ascites, coarse features, hepato-splenomegaly, inguinal hernia	Psychomotor retardation	n/r	Dysostosis multiplex	n/r	n/r	Cardiac anomalies	n/a	At the age of 2 months, patient was alive
Schmidt, M. [33]	F	Hydrops, ascites, edema, hepato-splenomegaly	Seizures	n/r	n/r	n/r	present	n/r	n/r	Exitus at 5 months
Ovali, F. [34]	M	Hydrops, ascites, coarse features, hepato-splenomegaly, inguinal hernia	n/r	n/r	n/r	n/r	present	n/r	n/r	Exitus at 27 days
Sergi, C. [35]	M	Hydrops, ascites, coarse features, hepato-splenomegaly, inguinal hernia	n/r	Corneal clouding	n/r	n/r	n/r	n/r	n/r	Exitus at 28 days
Sergi, C. [36]	M	Hydrops, edema ascites, hepato-splenomegaly	n/r	n/r	n/r	n/r	present	n/rn/r	n/r	Exitus at 2 months
Buchholz, T. [37]	M	Hydrops, edema ascites, hepato-splenomegaly	n/a	n/a	n/a	present	n/a	Cardiac anomalies	Telangiectasia Hypotonia	Exitus at 82 days
Uhl, J. [38]	M M	Hydrops, edema, ascites in both patients	n/a	n/a	n/a	n/a	n/a	n/r	Polydactyly in patient 1	n/a
Donati, M.A. [39]	F	Hydrops, ascites, edema, coarse features, hepato-splenomegaly, Inguinal hernia	Psychomotor retardation, Hydrocephalus	yellow/rretina	Dysostosis multiplex	present	present	Cardiac anomalies	Telangiectasia Hypotonia, Petechiae	Exitus at 19 months
Penzel, R. [40]	F	Hydrops, ascites, edema	Seizures	n/r	n/r	n/r	n/r	n/r	n/r	n/r
Itoh, K. [41]	M	Hydrops, ascites, edema, hepato-splenomegaly	n/r	n/r	n/r	n/r	n/r	n/r	n/r	Exitus at 27 days
Rodriguez Criado, G. [42]	M	Hydrops, coarse features, hepatosplenomegaly	Psychomotor retardation	n/r	Dysostosis multiplex	n/r	n/r	Cardiac anomalies	Hypotonia	Exitus at 20 months
Pattison, S. [43]	n/r	n/r	n/r	n/r	n/r	n/r	n/r	n/r	n/r	Exitus at Patient 1; 3 months Patient 2; 2 months
Loren, D.J. [44]	n/r	Hydrops, ascites, edema, hepato-splenomegaly	n/r	n/r	no	n/r	n/r	n/r	n/r	Alive at the age of three months
Caciotti, A. [22]	F	Coarse features, hepatosplenomegaly	Psychomotor retardation	n/r	Dysostosis multiplex	n/r	present	Cardiac anomalies	Telangiectasia Hypotonia, petechiae	Exitus at 1 year
Bonten, E.J. [7]	F	Hydrops, hepato-splenomegaly	Psychomotor retardation, Hydrocephalus	No corneal opacity	Dysostosis multiplex, Joint contractures	n/r	n/r	cardiomyopathy	Cardiac anomalies	Exitus at 18 months
Lee, Y.J. [45]	F	Hydrops, ascites, coarse features, hepatosplenomegaly	n/r	Bilateral congenital cataracts with foveal hypoplasia	n/r	n/r	n/r	n/r	Telangiectasia Hypotonia, bluish to purpuric macules mild thrombocytopenia	Exitus a t9 months
Lee, B.H. [46]	F	Hydrops, ascites, coarse features, edema, hepato-splenomegaly	n/r	n/r	n/r	Present	n/r	Cardiomegaly with huge patent ductus arteriosus (PDA), Ventriculomegaly	Hypotonia	Exitus a t3 months

**Table 2:** Clinical and Morphological findings of patients suffering from sialidosis II, subtype infantile/Juvenile form (Cases from 1980 until the date of submission are included). M = male; F = female; n/a = not available n/r = not reported; no = not detected; ECG = electrocardiogram; ** Authors reported the case “suspected as sialidosis II” after other congenital errors of metabolism investigated during her childhood, such as mucopolysaccharidosis, were excluded. No formal genomic testing is reported in the study.

References (Name of the First Author)	Gender	General Presentation	Nervous System	Ophthalmologic Findings	Skeleton	Respiratory Distress/Infections	Renal Involvement	Cardiac Involvement	Others	Course of Disease
Winter, R.M. [47]	M <1 year	Coarse feature	Psychomotor delay, Seizures	Visual loss	Dysostosis Multiplex	n/r	n/r	n/r	Hearing loss, Inguinal hernia	22 years
Kelly, T.E. [23]	F <1 year	Coarse feature, Hepatosplenomegaly	Psychomotor delay, seizures/myoclonic jerks	Cherry red spot	Dysostosis Multiplex	present	n/r	present	Umbilical hernia	5 and half years
Kelly, T.E. [23]	F Birth	Coarse feature, Hepatosplenomegaly	Psychomotor delay	Cataract	Dysostosis Multiplex	n/r	n/r	present	Hearing loss, Umbilical hernia, Hypotonia	24 months
King, M. [48]	M 5 months	Coarse feature, Hepatosplenomegaly	Psychomotor delay Ataxia	Cherry red spot, corneal Clouding, Cataract,	Dysostosis Multiplex	n/r/	n/r	n/r	Hearing loss	13 years
King, M. [48]	F N/A	Coarse feature, Hepatosplenomegaly	Psychomotor delay	Cherry red spot, Cataract,	Dysostosis Multiplex	n/r	n/r	n/r	Hearing loss	12 years
Oohira, T. [49]	F <1 year	Coarse feature, Hepatosplenomegaly	Psychomotor delay, Ataxia, myoclonic jerks	Cherry red spots	Dysostosis Multiplex	n/r	n/r	n/r	Hypotonia	5 years
Young, I.D. [50]	M 18 months	Coarse feature	Psychomotor delay, Ataxia, myoclonic jerks	Cherry red spot, Nystagmus, Optic atrophy	Dysostosis Multiplex	nr	n/r	n/r	Hearing Loss, Hypotonia	12 years
Bakker, H.D. [51]	F 6 months	Coarse feature	Psychomotor delay	Strabismus, Nystagmus	n/r	n/r	n/r	n/r	Hearing Loss, Hypotonia	30 years
Rodriguez Criado, G. [42]	M <1 year	Coarse feature, Hepatosplenomegaly	Psychomotor delay, Myoclonic movements Ataxia	n/r	Dysostosis Multiplex	n/r	n/r	present	Hearing Loss, Hypotonia	13 years
Rodriguez Criado, G. [42]	M 16 months	Coarse feature, Hepatosplenomegaly	Psychomotor delay	n/r	Dysostosis Multiplex	n/r	n/r	absent	Hearing Loss, Hypotonia	11 years
Pattison, S. [43]	n/r	Coarse feature, Hepatosplenomegaly	n/r	n/r	Dysostosis Multiplex	n/r	n/r	n/r	n/r	3 years
Pattison, S. [43]	n/r	Coarse feature, Hepatosplenomegaly	n/r	n/r	Dysostosis Multiplex	n/r	n/r	n/r	n/r	3 years
Schiff, M. [52]	F <1 year	Coarse feature, Hepatosplenomegaly	Psychomotor delay	n/r	Dysostosis Multiplex	n/r	present	n/r	n/r	11 years
Gonzalez Gonzalez G [53]	n/r	n/r	Myoclonic epilepsy	n/r	Dysostosis Multiplex	n/r	n/r	present	n/r	14 years
Caciotti, A. [22]	M 1 year	Coarse feature	Psychomotor delay Seizures	Cherry red spot, cataract,	Dysostosis Multiplex	n/r	n/r	n/r	Hearing Loss	9 years
Bonten, E.J. [7]	M birth	Coarse feature, Hepatosplenomegaly	Developmental delay, Orbital hypoplasia	normal	Craniosynostosis	n/r	n/r	n/r	n/r	Progressing at 4 months
Bonten, E.J. [7]	F 12 years	n/r	Psychomotor delay, Seizures Ataxia, Dysmetria Spasticity	Cherry red spots	Dysostosis Multiplex, microcephaly	n/r	n/r	ECG specific alterations of repolarization	Hearing Lossl	Progressing at 28 years.
Ranganath, P. [54]	F 18 months	Coarse facies, hepatomegaly	n/a	Mild corneal haziness, bilateral fundal Cherry red spots	Macrocephaly	n/r	n/r	Cardiac anomalies	Protuberant tongue, gum hypertrophy, generalized hypertrichosis, large Mongolian spots on the back, umbilical hernia	n/r
de Rezende Pintoi ** [55]	F 30 years	a high forehead and low-set ears	Advanced degree of mental deficiency.lower limb spasticity, and facial and limb myoclonic jerks	Bilateral macular cherry-red spots	n/r	n/r	n/r	n/r	A marked delayed in motor and cognitive functions present since childhood. Cognitive and motor skills had worsened over 10 years	n/r
